# A prospective, randomized, double-blinded single-site control study comparing blood loss prevention of tranexamic acid (TXA) to epsilon aminocaproic acid (EACA) for corrective spinal surgery

**DOI:** 10.1186/1471-2482-10-13

**Published:** 2010-04-06

**Authors:** Kushagra Verma, Thomas J Errico, Kenneth M Vaz, Baron S Lonner

**Affiliations:** 1 Department of Orthopaedic Surgery, NYU Hospital for Joint Diseases - Langone Medical Center, 301 East 17th St, New York, NY 10003 USA

## Abstract

**Background:**

Multilevel spinal fusion surgery has typically been associated with significant blood loss. To limit both the need for transfusions and co-morbidities associated with blood loss, the use of anti-fibrinolytic agents has been proposed. While there is some literature comparing the effectiveness of tranexamic acid (TXA) to epsilon aminocaproic acid (EACA) in cardiac procedures, there is currently no literature directly comparing TXA to EACA in orthopedic surgery.

**Methods/Design:**

Here we propose a prospective, randomized, double-blinded control study evaluating the effects of TXA, EACA, and placebo for treatment of adolescent idiopathic scoliosis (AIS), neuromuscular scoliosis (NMS), and adult deformity (AD) via corrective spinal surgery. Efficacy will be determined by intraoperative and postoperative blood loss. Other clinical outcomes that will be compared include transfusion rates, preoperative and postoperative hemodynamic values, and length of hospital stay after the procedure.

**Discussion:**

The primary goal of the study is to determine perioperative blood loss as a measure of the efficacy of TXA, EACA, and placebo. Based on current literature and the mechanism by which the medications act, we hypothesize that TXA will be more effective at reducing blood loss than EACA or placebo and result in improved patient outcomes.

**Trial Registration:**

ClinicalTrials.gov ID: NCT00958581

## Background

Multilevel spinal fusion surgery has typically been associated with significant blood loss and transfusion requirements. Significant patient factors affecting operative blood loss include duration of exposure, severity and type of spinal deformity, and patient height [1-3]. Surgery dependent factors include operating time, procedure performed, combined anterior/posterior approaches, number of vertebrae fused, number of anchors placed, average mean arterial pressure (MAP) during surgery, blood salvage techniques, and the use of anti-fibrinolytic medications [4]. Factors with an unclear role include, but are not limited to: pre-operative hemoglobin, autologous donation, history of coagulopathy, prior use of anticoagulant medication, and type of fibrinolytic medication used in the operating room.

Large quantities of intra-operative and postoperative blood loss require blood transfusion to maintain tissue perfusion and prevent end-organ damage. The use of allogenic blood, however, confers an additional risk for blood borne pathogens. Also noteworthy is the risk for transfusion related reactions, immune suppression, and a decrease in coagulation factors. There is also evidence that transfusion of allogenic blood is increasingly harmful as more blood is transfused [5]. While the innovation of autologous transfusion, cell-salvage, and pre-operative erythropoietin administration has reduced the need for allogenic transfusion, patients undergoing spinal fusion may lose up to their entire blood volume or more for highly complex spinal reconstructive procedures [6].

More recently the use of anti-fibrinolytics has come into favor for cardiac and orthopedic surgery where blood loss is of significant concern. These include aprotinin, tranexamic acid (TXA), and epsilon aminocaproic acid (EACA, trade name Amicar?). Aprotinin is a serine protease inhibitor with anti-fibrinolytic properties. In contrast, TXA and EACA are synthetic lysine analogs that act as inhibitors of fibrinolysis. TXA is ten times more potent than EACA and binds more strongly to the plasminogen molecule [7].

The safety of these treatments has been studied in the orthopedic and cardiac literature [8]. Aprotinin lost FDA approval due to safety concerns [9]. However, both TXA and EACA have excellent safety profiles and continue to be used widely by many institutions. In the spine literature, there continues to be a paucity of data evaluating the optimal guidelines or indications for administration of TXA and EACA. No studies offer level-1 evidence comparing the efficacy of both treatment options head-to-head.

## Methods/Design

### Trial Organization

This trial will be conducted at single institution, New York University-Langone Medical Center.

### Investigators

All patients are recruited through both the private offices of the investigators and clinic of New York University - Hospital for Joint Diseases. Patients are consented by the investigators, persons completing research fellowships in orthopaedic surgery and/or persons working exclusively on clinical orthopaedic research projects. These researchers all have experience seeing and consenting patients.

### Medication Supply and Randomization

On the day of surgery, the anesthesiologist orders either TXA or EACA from the pharmacy. Orders for both medications are placed onto a single order form with both the medical registration number and the patient's de-identified identification number (ID). Prior to study, patient de-identified ID numbers were randomly assigned to the TXA, EACA, or placebo (1 to 150) using computer generated random assignment. Since additional patients with neuromuscular scoliosis (NMS) and adult deformity (AD) were later included into the study, supplementary de-identified ID numbers were added to the original 150 consecutive de-identified ID numbers. In a similar fashion, these consecutive de-identified ID numbers (151 to 390) were randomly assigned to TXA, EACA or control. The updated list of de-identified ID numbers and their respective treatment groups (TXA, EACA, or Control) were also placed in a sealed envelope and made available to the physicians, anesthesiologists, and residents involved with patient care. A copy was also given to the pharmacy department and to the researchers in a sealed envelope. The list of de-identified ID numbers and their treatment groups will remain unchanged for the duration of the study. Based upon the identification number and the randomization list, the pharmacist prepares TXA, EACA, or placebo using the methods listed in this protocol.

### Physician Blinding

While clinicians are blinded to the patient's particular treatment group, at any time during surgery or in the post-operative period any clinician can be "unblinded" to the patient's treatment group if there is a concern for a complication. In addition, all patients are followed daily by the residents and fellows on the spine service and will also be followed by spine research fellows caring for the patient. The research residents also record and document basic metabolic profile (BMP) and complete blood count (CBC) lab values daily. The research fellows pay careful attention to the blood creatinine and blood urea nitrogen/creatinine ratio (BUN/Cr) monitoring daily for possible renal failure. Sequential compression devices (SCD) are given to all spine patients and they are encouraged to mobilize out of the hospital bed on post-op day 2. Spine patients are closely monitored for signs of a DVT by the medicine attending physicians and nurses caring for patients.

The anesthesiologist, surgeon, and researcher can be unblinded from the study at any time in the case of an apparent or suspected medical emergency. A copy of the consent form is placed in every chart in addition to a list of contact numbers of research persons involved with the study. There is also a note on the front cover of the chart informing all nurses and physicians that the patient is enrolled in the study with contact information for the research fellows and for the pharmacy department. The pharmacy is instructed to release the treatment group for any patient if requested by medical personnel caring for the patient or a spine research fellow. Order forms given to the pharmacy will be kept in a secure binder and will be made available upon request. As an additional resource, all physicians, anesthesiologists, and residents involved with patient care will have access to a sealed envelope with patient de-identified ID numbers and their assigned treatment groups.

### Adverse events management

Complications include but are not limited to: suspected medication or allergic reaction, suspected myocardial infarction, stroke, deep vein thrombosis, pulmonary embolism, and renal failure. Other side effects that have been reported have been minor including headache, upset stomach, flushing, and hypotension. To date there has been no clear association between the use of anti-fibrinolytics, such as TXA and EACA, and renal failure or thrombosis [7,8]. A similar medication with anti-fibrinolytic properties, aprotinin, did lose FDA approval over concerns regarding renal failure in cardiac patients and the theoretical risk of thrombosis [9]. Aprotinin functions as a serine protease inhibitor. In contrast, TXA and EACA are synthetic lysine analogs that bind to and produce a structural change in plasminogen preventing conversion of plasminogen to plasmin and conversion of fibrinogen to fibrin. TXA and EACA therefore have a dual mechanism of anticoagulation: decreased fibrinolysis and decreased platelet aggregation due to the inactivation of plasmin. This difference in mechanism most likely accounts for the lack of association between the use of anti-fibrinolytics and renal failure [7,8]. Regardless, patients in the study are carefully monitored for signs of renal failure post-operatively. Any patient with an elevated or rising creatinine is carefully followed by resident and attending orthopaedic surgeons, internal medicine physicians, and the research fellows. If the elevation in creatinine is suspicious for renal failure, patients will be "unblinded" and treated appropriately. TXA and EACA have been used most in cardiac and spinal surgery and meta-analysis has clearly shown a reduction of intra-operative blood loss and allogenic transfusion rate with these medications [6-8,10,11,13].

Considering the reported reduction in operative blood loss and allogenic transfusion rate in both the cardiac and spine literature, the benefits of treatment outweigh the potential side effects. While these medications are widely used, they are currently not the standard of care in spinal surgery. This study aims to delineate the benefits of TXA versus EACA in spinal surgery, which may help to identify the patients most in need of this treatment. Complications will be diligently recorded and reported in the final manuscript.

### Ethics, informed consent, and safety

The Institutional Review Board (IRB) of NYU approved the research protocol. If the patient wishes to enroll in the study, the IRB approved informed consent process is undertaken. A copy of the signed informed consent is issued to the family during the pre-operative office visit. The consent form includes the purpose of the study, a description of the study, costs and reimbursements, potential risks and discomforts as well as the primary investigators' contact information. If the patient is a minor, consent is obtained from the guardian of the minors using an NYU IRB approved consent form. Consent is also obtained from minors using an NYU IRB approved consent form.

Consented individuals are informed of the use of anti-fibrinolytic medication to control intra-operative and postoperative bleeding. The risks, benefits, advantages, and disadvantages of the use of TXA versus EACA versus placebo to control blood loss are detailed with the patient and family, as part of their routine pre-operative visit with their surgeon. The patient and family are diligently informed that while TXA and EACA have individually been shown to reduce total blood loss, neither medication is recognized as the standard of care. The relative affect of these medications with relation to transfusion rate and patient outcomes is also not fully known.

### Patient selection

The study concerns patients undergoing thoracic and/or lumbar spinal surgery for the correction of adolescent, idiopathic scoliosis (AIS), neuromuscular scoliosis (NMS), and adult deformity (AD). Inclusion and Exclusion criteria are included in Table 1.

**Table 1:** Inclusion and Exclusion Criteria

All Participants	
Inclusion	Exclusion
1. Undergoing thoracic and/or lumbar surgery for correction of condition via posterior spinal fusion of greater than 6 levels	1. No renal dysfunction identified by elevated blood urea nitrogen (BUN) and creatinine (CR) or BUN to CR ratio greater than 20:1
	2. Hold religious and/or other beliefs limiting blood transfusion
	3. Currently use anti-coagulant medication or have past medical history leading to abnormal coagulation profile pre-operatively
	4. Significant past medical history preventing the use of TXA or EACA described in the protocol
**AIS**	
Inclusion	Exclusion
1. Between 10 and 21 years of age	1. Previous corrective spinal surgery that is being revised
2. BMI between 5th and 95th percentiles	
**AD**	
Inclusion	Exclusion
1. Between 18 and 80 years of age	1. Any history of coronary artery disease with stent placement
**NMS**	
Inclusion	Exclusion
1. Between 10 and 80 years of age	1. Congenital and syndromic scoliosis
	2. Previous corrective spinal surgery that is being revised

The maximum total number of patients enrolled will be 150 of AIS (50 in each treatment arm), 90 for NMS (30 in each treatment arm), 150 for AD (50 in each treatment arm). These numbers were estimated by critically reviewing the existing spine and cardiac literature comparing TXA and EACA against placebo [6-20]. A power analysis was conducted using the literature to determine the number of patients necessary to attain significant results (Tables 2 and 3).

**Table 2:** Power study to determine number of patients needed in AIS group.

	TXA		Amicar		Control		
	**Blood loss**	**Standard Deviation**	**Blood Loss**	**Standard Deviation**	**Blood Loss**	**Standard Deviation**	**Sample Size Needed**

Intra-op blood loss	1072 mL	+/- 425 mL	-	-	1420 mL	+/- 644 mL	58

Intra-op blood loss	1072 mL	+/- 425 mL	893 mL	+/- 220 mL	-	-	80

Total blood transfused	1253 mL	+/- 884 mL	-	-	1784 mL	+/- 733 mL	55

Total units transferred	-	-	1.2 U	+/- 1.1 U	2.2 U	+/- 1.3 U	34

**Table 3:** Power study to determine number of patients needed for NMS group

	TXA		Amicar		Control		
	**Blood loss**	**Standard Deviation**	**Blood Loss**	**Standard Deviation**	**Blood Loss**	**Standard Deviation**	**Sample Size Needed**

Intra-op blood loss	1408 mL	+/- 605 mL	-	-	2690 mL	+/- 1266 mL	14

Intra-op blood loss	1976 mL	+/- 860 mL	1117 mL	+/- 718 mL	-	-	30

Total blood transfused	808 mL	+/- 531 mL	-	-	1391 mL	+/- 723 mL	34

### Study Design

The study is designed as a prospective, randomized, double-blinded control trial. The patient, researcher, surgeon, and anesthesiologist will all be blinded to the patient's treatment. Patients will either receive TXA, EACA, or normal saline. The study will determine and compare differences in blood loss intra-operatively and estimate blood loss postoperatively through monitoring of subfascial Hemovac drain outputs at the incision site between the three groups. Postoperatively, laboratory values, drain outputs, and clinical outcomes will be carefully followed.

The primary goal of this study is to compare the efficacy of TXA to EACA to placebo in patients undergoing corrective spinal surgery for adolescent idiopathic scoliosis (AIS), neuromuscular scoliosis (NMS), and with adult deformity (AD). Outcome measures will include intra-operative and perioperative blood loss, transfusion rates, complete blood counts, and coagulation profiles, as well as postoperative wound drainage, complications, and length of stay.

### Standardized treatment protocol

The infusion is started 15 minutes prior to incision. The infusion is given in two parts. First a loading dose is given over 15 minutes at a volumetric rate of 20 ml/hour for a 50 kg patient. Following the initial loading dose, a maintenance dosage is given at one-tenth of the loading dose or 2 ml/hour for a 50 kg patient. This volumetric rate is adjusted accordingly for the patient's body weight. The volume infused remains constant regardless of whether the patient is receiving TXA, EACA, or saline. TXA is administered at 10 mg/kg hr for a loading dose followed by 1 mg/kg hr for a maintenance dose. In contrast, EACA is administered at 100 mg/kg hr for a loading dose, followed by 10 mg/kg hr for a maintenance dose. To adjust for this difference, the pharmacy is instructed to prepare the EACA at a tenfold higher concentration (250 mg/ml) than the TXA (25 mg/ml). The TXA and EACA treatments are both prepared in saline and both treatment preparations are visually indiscernible from saline alone. The loading and maintenance doses for TXA and EACA used in the protocol are in accordance with recent literature citing efficiency and safety at these dosages [7,8,10-14]. The literature also reports safely using TXA at a 100 mg/kg loading dose followed by a 10 mg/kg hr maintenance dose. However, improved efficacy at this higher dose of TXA has not been proven in a single orthopedic study. Since TXA is reported to be ten times more potent than EACA, delivering a dose of TXA that is ten times smaller than EACA is valid and well supported in the literature.

### Surgical correction of the spine

Dorsal lumbar access is achieved by a posterior lumbar skin incision along the midline. Paravertebral muscles are dissected away from bony structures taking care to achieve adequate hemostasis. Multi-level spinal osteotomies and/or releases of bony and ligamentous structures are performed to increase mobility of the spinal column. Bone grafts consisting of autologous bone or allograft are filled in to allow for bony fusion of the spinal column and limit motion. Pedicle screws are placed segmentally into vertebral bodies to provide adequate fixation of rod instrumentation. Stainless steel, titanium, or cobalt rods are placed to help correct and stabilize the spine while fusion occurs. Generally the greatest blood loss occurs during dissection and placement of pedicle screws while rod placement is associated with less blood loss. Subfascial Hemovac or Jackson-Pratt drains are placed at the wound site during closure to allow for adequate drainage and determination of post-operative blood loss.

### Investigation schedule and follow-up

Data will be collected pre-operatively regarding individual patient demographics, laboratory values, and the surgical procedure to be performed. Intra-operatively, data will be gathered to estimate blood loss and account for changes in fluid balance. Anesthesiologists are asked to maintain a MAP of 60 during the surgical exposure and anchor placement and a MAP of 70-90 during the surgical correction. Similarly, surgeons are asked to place only subfascial Hemovac drains at the incision site. These are both standard practice and have been shown by our group to help control blood loss during surgical exposure [21]. Postoperatively, laboratory values, drain output, and clinical outcomes are carefully followed until the patient is discharged. Given the increased risk of renal failure with the use of Aprotinin [7] changes in BUN to CR ratio are carefully monitored post-operatively. This data is recorded onto a de-identified data collection sheet by the researchers. These patient data sheets are then entered onto a protected electronic database, while the data sheets are stored as a back-up until the study is complete. Once the completed database is analyzed and summarized, the results will be presented to the involved participants without any identifiable patient information.

### Statistical considerations

While useful, this analysis is limited by the great variability in study design and control groups between prior orthopedic studies. Since no orthopedic study has compared TXA and EACA together, conducting a power analysis required pooling of data from two or three studies. This proved difficult, as control groups were dissimilar between the studies (Table 2).

Power analysis was performed multiple times using data from several studies. When estimating the sample size required comparing TXA and EACA, attempts were made to choose two studies with similar intra-operative blood losses within the control groups. A cardiac study, albeit with dissimilar sample variance, was also used to estimate the sample size needed for this study. Finally, determining the sample size for outcomes of total blood loss and transfusion requirements proved difficult as even fewer studies reported these outcomes. Blood loss will be estimated in the operation room, but will also be calculated taking into account patient body mass, pre/postoperative hematocrit, and relative fluid balance [21-23]. This will minimize the variance in reported blood loss. A power analysis will be performed again once 60 patients with AIS, NMS, or AD have been enrolled into the study.

With a single institution of surgeons and anesthesiologists participating in the study, we expect a smaller variation in operative blood loss than reported in prior studies. Data will not only be analyzed for all patients collectively, but will also be stratified by diagnosis (AIS, NMS, and AD). The purpose of stratifying into these groups is threefold. First, separation by diagnosis allows for more diligent control of patient-related factors that may affect blood loss and transfusion rate. Secondarily, stratification by diagnosis allows for a better understanding of which patients benefit most from one treatment option over another. Lastly, it allows for a broader application of the results from this study not only to patients with AIS, but to all spinal deformity patients.

An analysis of variance, univariate, and multivariate logistic regression analysis will be used to analyze the difference in outcomes. Odds ratios will be calculated regarding the risk for autologous or allogenic transfusion both intra and postoperatively. P-values will be calculated regarding the relative blood loss in the intra and postoperative periods as well. The groups will be analyzed to characterize the homogeneity of their pre-operative characteristics that may influence blood loss. Patients with AIS, NMS or AD will be stratified by primary diagnosis and analyzed separately but also collectively accounting for patient and surgery-related confounders. Non-continuous data will be analyzed with a non-parametric test. Pre-operative curve characteristics including Cobb angle and number of vertebrae fused will be categorized to ensure similar groups for comparison.

## Discussion

Overall, orthopedic studies have demonstrated the efficacy of TXA and EACA over placebo in relation to blood loss [8-16]. However, the specifics of this demonstrated benefit are lacking. In particular, a reduction in intra-operative blood loss has been inconsistently reported. In two recent Cochrane reviews, TXA was noted to be more efficacious than EACA at reducing total blood loss, but neither drug significantly reduced intra-operative bleeding [7,8]. The most recent review demonstrated a reduction in transfusion volume with antifibrinolytic medications in scoliosis surgery, but could not comment on the benefit of one medication over another [7,8]. In addition, studies solely reporting intra-operative blood losses may have overlooked postoperative benefits. The authors concluded that antifibrinolytic treatments have greatest efficacy in controlling postoperative bleeding, but also that more investigation was needed. Comparing trials in all surgical fields including cardiac surgery, TXA - but not EACA - was found to reduce the transfusion rate over placebo.

The cardiac literature has produced seven prospective studies comparing TXA and EACA head-to-head, but no trial was assessed as having proper patient randomization and physician blinding [7,18-20]. Moreover, for five of the seven trials the method of randomization raised uncertainty for the reviewers [7]. In addition, the trials were inappropriately powered to detect differences between TXA and EACA and excluded high-risk patients to accentuate differences between the two drugs.

The role of anti-fibrinolytics to manage blood loss, therefore, remains largely surgeon dependent and is not the standard of care in spinal deformity surgery. Of the scoliosis studies examined in the Cochrane review, only two studies adequately randomized patients and blinded both the surgeon and anesthesiologist to the patient's treatment [8]. Since operative blood loss is partly dependent upon surgical and anesthetic conditions, proper physician blinding is imperative. As stated, no single orthopedic study has prospectively compared the relative efficacy of TXA and EACA head-to-head.

The goal of this proposal is to perform a randomized, prospective, double blind study comparing the relative efficacy of TXA, EACA, and controls in AIS, NMS, and AD patients. AIS patients are particularly well suited to investigate because these patients are generally healthy, and free from use of anticoagulant medications. These factors all help to minimize the effect of known confounders effecting blood loss. Patients with NMS, however, have a heterogonous profile and often have multiple confounding medical problems that may affect blood loss. Typically these patients have significant larger blood loss and transfusion needs as compared to AIS patients. However, this difference may accentuate differences in blood loss for TXA versus EACA. Finally, AD patients represent the largest subgroup of spinal deformity patients. Their inclusion into this study allows for the results to have a broader application across spinal deformity patients.

We hope that this study will determine if a difference in efficacy exists between TXA, EACA and placebo and begin to establish a new standard of care in corrective spinal surgery. The use of patient populations beyond those of AIS may have meaningful results that can be applied to a wider range of patients undergoing corrective spinal procedures by reducing morbidities associated with blood loss.

## Competing interests

The authors declare that they have no competing interests.

## Authors' contributions

TE and BL were responsible for the concept of the study, editing, and final approval of this protocol. KV was responsible for conception of the study and study design, as well as writing, reviewing, editing, and data collection for this protocol. KMV was responsible for reviewing and editing. All authors read and approved the final manuscript.

## Pre-publication history

The pre-publication history for this paper can be accessed here:

http://www.biomedcentral.com/1471-2482/10/13/prepub
